# Does aerobic scope influence geographical distribution of teleost fishes?

**DOI:** 10.1093/conphys/coad012

**Published:** 2023-03-29

**Authors:** Julie J H Nati, Lewis G Halsey, Paul C D Johnson, Jan Lindström, Shaun S Killen

**Affiliations:** School of Biodiversity, One Health and Veterinary, Graham Kerr Building, Glasgow G12 8QQ, UK; Department of Life Sciences, University of Roehampton, Holybourne Avenue, London SW15 4JD, UK; School of Biodiversity, One Health and Veterinary, Graham Kerr Building, Glasgow G12 8QQ, UK; School of Biodiversity, One Health and Veterinary, Graham Kerr Building, Glasgow G12 8QQ, UK; School of Biodiversity, One Health and Veterinary, Graham Kerr Building, Glasgow G12 8QQ, UK

**Keywords:** teleost fish species, geographical distribution, ecophysiology, comparative physiology, Aerobic scope

## Abstract

Many abiotic and biotic factors are known to shape species' distributions, but we lack understanding of how innate physiological traits, such as aerobic scope (AS), may influence the latitudinal range of species. Based on theoretical assumptions, a positive link between AS and distribution range has been proposed, but there has been no broad comparative study across species to test this hypothesis. We collected metabolic rate data from the literature and performed a phylogenetically informed analysis to investigate the influence of AS on the current geographical distributions of 111 teleost fish species. Contrary to expectations, we found a negative relationship between absolute latitude range and thermal peak AS in temperate fishes. We found no evidence for an association between thermal range of AS and the range of latitudes occupied for 32 species. Our main results therefore contradict the prevailing theory of a positive link between AS and distribution range in fish.

## Teaser

Fishes with a large geographical distribution are expected to also have a large aerobic scope (AS). Our study of the literature found, however, a negative correlation between maximum AS and distribution range fishes living in temperate climates. However, no link was found between thermal range AS and latitudinal range in 32 species.

## Introduction

The geographical distributions of species are shaped by a multitude of interacting factors including temperature (Payne *et al*., 2018), intraspecific and interspecific interactions (Hansson, 1984) and dispersal capacity (Gaston, 2009; Kubisch *et al*., 2014). Another suite of influential traits are species' physiological responses to their environment (Kearney and Porter, 2009). We can therefore assume that biogeographical trends will covary with species' physiology (Somero, 2005; Bozinovic *et al*., 2011; Somero, 2011; Huey *et al*., 2012; Naya and Bozinovic, 2012; Seebacher *et al*., 2015). For ectotherms, aerobic scope (AS) has been proposed as a physiological constraint on species' geographic ranges (Pörtner, 2001; Pörtner and Farrell, 2008). AS represents the cardiovascular and respiratory capacity of an animal to perform oxygen-fueled processes (such as growth, locomotion and reproduction) above those required for basic maintenance (Fry, 1971; Clark *et al*., 2013). AS in ectotherms often increases with rising temperature, peaks at an optimum temperature and then decreases with further warming (Fry, 1971; Farrell, 2016; cf. Lefevre, 2016). An ambient temperature that differs substantially from an animal's AS thermal optimum could therefore lower that animal's capacity to deliver oxygen to tissues, which in turn may impose constraints on its ability to perform physical activity or invest energy in reproduction or growth (Pörtner, 2001).

**Figure 1:**
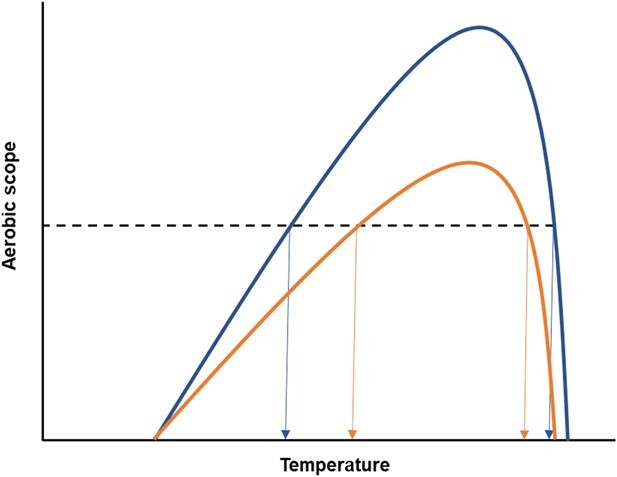
Theoretical relationship between aerobic scope and ambient temperature range, denoting the temperatures across which sufficient oxygen can be allocated to all necessary aerobic processes for the body to function effectively (‘high aerobic scope’; dashed line). A species with a high peak AS (blue line) may be able to function effectively over a larger thermal range (blue arrows) (termed a greater thermal breadth of AS) than a species with a low peak AS (orange line and arrows). Figure modified from Nati *et al*., 2016.

Recently, it has been proposed that a relatively high AS may accommodate a high capacity for behavioural and physiological plasticity (Biro *et al*., 2018). We therefore hypothesise that species with a high thermal peak AS, or that exhibit a high AS over a large range of temperatures, have the plasticity required to survive over a broad range of thermal environments. Indeed, often, such species may present both a high peak AS and a great thermal breadth of high AS. This is because there appears to be no functional trade-off between peak AS and breadth of high AS across temperatures, at least for teleost fishes (Nati et al., 2016), and thus species with a high peak AS may have an AS that remains high across a broad temperature range (Fig. 1). We therefore predict a positive correlation between peak AS and thermal breadth of high AS with latitudinal distribution.

Several studies on endotherms and terrestrial ectotherms have demonstrated positive relationships between metabolic traits and geographic distributions (Rezende *et al*., 2001, 2004; Dillon *et al*., 2010; Naya *et al*., 2012). For example, a positive correlation between AS and latitude was reported across 48 rodent species (Naya *et al*., 2012). For ectotherms, such as fish, physiology is more dependent on ambient environmental conditions and therefore could strengthen the correlation between AS and geographical distribution. To date, however, no broad comparative study in an ectothermic taxon, such as teleost fishes, has investigated the links between AS and species distribution. Most studies investigating relationships between metabolic traits and distribution in fishes have focused on a few species and over relatively narrow latitudinal gradients (Gardiner *et al*., 2010; Rummer *et al*., 2014; Payne *et al*., 2016). For example, in a four-species comparison of coral reef fish, Gardiner *et al*. (2010) showed that populations of each species at a high latitude had a greater AS than populations at a lower latitude. Over a broader geographical scale, across 38 fish species, a positive correlation was demonstrated between latitude range and metabolic scope (Naya and Bozinovic, 2012). This analysis, however, did not account for phylogenetic relatedness and defined metabolic scope as being the difference between routine metabolic rate (RMR) and standard metabolic rate (SMR). This definition does not correspond to AS but, depending on how RMR is defined for each study, may approximate daily routine energy expenditure. Payne *et al*. (2016) found a positive relationship between the optimum temperature for AS and the highest temperature encountered in the geographical range of 12 tropical and temperate marine fishes, suggesting that AS might set the upper thermal limit for geographical regions that are habitable by these species. Therefore, AS may well be relevant for determining the upper distribution boundaries in teleost fishes. It is in part due to these findings that there is an acceptance in the literature that aerobic performance in ectotherms, such as fish, is positively related to their capacity to occupy a wider range of different thermal environments (e.g. Pörtner, 2001; Pörtner and Farrell, 2008; Farrell, 2016), but the current evidence for this is far from compelling (Nati *et al*., 2016).

The climatic variability hypothesis (CVH) states that animals at higher latitudes experience greater thermal fluctuations and therefore display broader thermal performance breadths and high physiological plasticity (Janzen, 1967; Stevens, 1989; Bozinovic *et al*., 2011). This suggests that temperate species have broader thermal tolerance ranges than tropical species. Although the CVH has been examined in several ectothermic taxa (e.g. *Drosophila* spp., Overgaard *et al*., 2011; insects, Addo-Bediako *et al*., 2000; lizards, Deutsch *et al*., 2008), it seems to apply only to terrestrial species (Bozinovic *et al*., 2011). In addition, Rapoport's rule states that the range size of species decreases towards the equator (Stevens, 1989), suggesting that tropical species would have narrower distribution ranges than temperate species. However, it is unclear whether this rule applies to teleost fishes (Rohde *et al*., 1993) and how the implications of Rapoport's rule interact with species' metabolic physiology. A greater understanding of how temperate and tropical species may have evolved different thermal breadths for physiological performance or physiological plasticity would provide insight into how each of these two thermal groups may be able to respond to a changing global climate.

**Table 1:** Summary of the PGLS model testing for the effects on absolute latitude range (0–90°) of peak aerobic scope (log_10_ AS (mg O_2_ h^−1^)), mass (log_10_ mass in g), acclimation temperature (°C), lifestyle (benthic, benthopelagic or pelagic), behaviour (migratory or sedentary), thermal group (tropical or temperate), habitat (marine, freshwater or combination marine/freshwater) and the interaction term between thermal group and log_10_ AS. *R^2^* = 0.363, F_13, 96_ = 4.215, p < 0.0001, n = 111 species, λ = 0.00, d.f. = 96. For lifestyle categorisation, the reference category is ‘benthic’; for behaviour it is ‘migratory’; for thermal group it is ‘tropical’; for the interaction it is ‘tropical’; for habitat it is ‘freshwater’

Term	Estimate	s.e.	*t*	*p*
Intercept	30.87	4.31	7.33	< 0.001
log_10_ AS	−1.43	4.06	−0.35	0.725
log_10_ mass	3.802	3.61	1.05	0.294
Temperature	0.002	0.24	0.008	0.99
Lifestyle				
Benthopelagic	0.46	2.69	0.171	0.87
Pelagic	4.951	4.11	1.20	0.232
Reef-associated	−8.962	4.63	−1.94	0.056
Behaviour				
Sedentary	−3.03	2.74	−1.106	0.272
Thermal group				
Temperate species	−3.22	3.54	−0.909	0.366
Polar species	−5.325	5.77	−0.92	0.36
Habitat				
Marine species	14.07	3.22	4.37	<0.001
Marine/freshwater	10.26	3.32	3.09	0.003
log_10_AS:thermal group				
Temperate species	−5.48	2.26	−2.42	0.02
Polar species	3.52	7.85	0.448	0.655

Teleost fishes are a diverse ectothermic taxon found in variable aquatic habitats. They have vastly differing geographical ranges and lifestyles, with some species being much more active than others. Furthermore, there is now a considerable amount of data available on fish metabolic traits. Therefore, teleost fishes are a suitable study model to test our main hypothesis (Fig. 1). We propose that a bigger/higher thermal peak of AS and wider thermal breadth of AS in fishes will associate with them having larger geographical ranges. In the current study, we focused on using interspecific, phylogenetically informed analyses to examine relationships between AS and latitudinal distribution range of 111 teleost fish species. We investigated whether latitudinal distribution range of fish species is associated with, and therefore potentially determined by, peak of AS and thermal breadth of AS. The results presented here provide insight into the degree of influence of AS on current species distributions.

**Figure 2:**
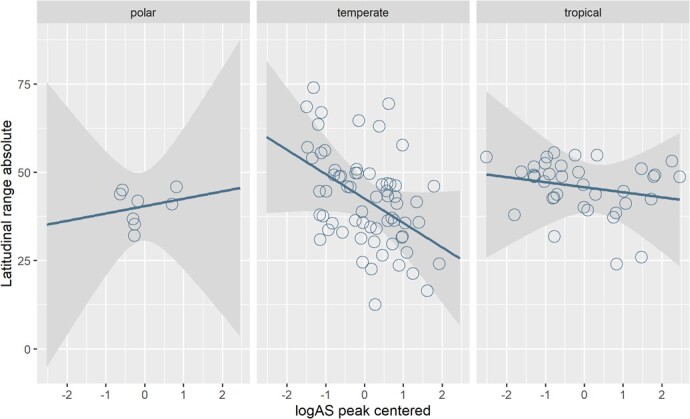
Relationship between absolute latitude range occupied by each species (0–90°) and log_10_ peak aerobic scope (log_10_ AS mg O_2_ h^−1^) centred for 111 fish species, categorised as temperate (n = 66), tropical (n = 37) or polar (n = 8). Each data point represents a distinct species.

**Figure 3:**
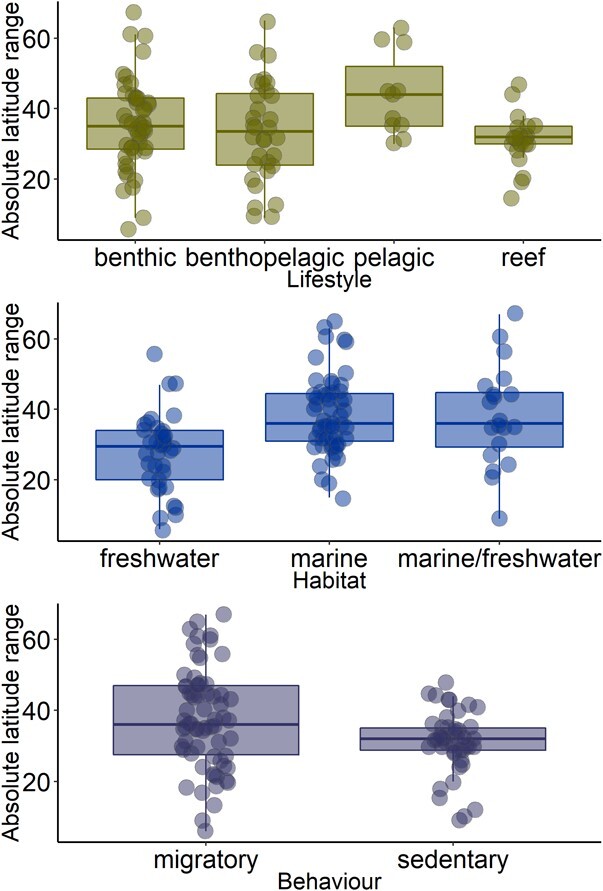
Absolute latitudinal range categorised by (a) lifestyle (benthic = 47, benthopelagic = 32 and pelagic = 11, reef-associated = 21 species), (b) habitat type (freshwater = 36, marine = 55, marine/freshwater = 20 species) and (c) behaviour (migratory = 67, sedentary = 44 species). Each data point represents a distinct species.

## Methods

### Data collection

#### (a) Metabolic rate data and calculating peak aerobic scope

Data on the standard and maximum metabolic rates of 116 species of fish were collated from the literature (see suppl. data set). SMR (mg O_2_ h^−1^) is defined as the minimum energy required to sustain life, and maximum metabolic rate (MMR; mg O_2_ h^−1^) represents the maximum rate of aerobic metabolism that an animal is able to perform. Whole animal absolute aerobic scope (AS; mg O_2_ h^−1^) was calculated as the difference between MMR and SMR. To collate these data, we searched Web of Knowledge and Google Scholar using keywords ‘standard metabolic rate’ or ‘maximum metabolic rate’ or ‘aerobic scope’ and ‘fish’. Species were only included when both SMR and MMR were measured in the same study. For SMR, we only considered studies which measured metabolic rates as follows: 1) by extrapolating oxygen consumption values measured at different activity levels to zero activity level; or 2) by direct recording of oxygen rates of a post larval, starved and resting fish over a consistent period of time; for MMR: 1) by recording critical peak oxygen rates during forced swimming in a swim tunnel, or 2) by measuring oxygen consumption immediately after exhausting exercise in a swim tunnel or 3) after a chasing protocol (Clark *et al*., 2013). These methods are known to provide similar values of MMR at the species level (Killen *et al*., 2017). Where we found several studies for the same species, we selected one data set per species according to the following criteria. 1) For studies in which differing size classes were measured, we used the study which collected data using fish with the greatest body mass to select for juvenile or adult stages. These life stage categories are the less thermally sensitive stages in fishes (Pörtner and Farrell, 2008). Our mass range was from 0.05 to 6450 g. 2) For studies in which multiple temperatures were examined, we used the study that measured metabolic rates at the greatest number of acclimation temperatures. Our acclimation temperature ranged from 3°C to 35°C. Besides collecting metabolic rates (*ṀO_2_*) from each study, we also recorded the mean body mass (g) of the fish and the acclimation temperature used in each study. In cases where *ṀO_2_* was measured at more than one temperature (studies with one acclimation temperature, n = 58; more than one acclimation temperature, n = 53), we used the data at the acclimation temperature for which AS was highest. However, as most studies measured AS at one temperature, we cannot presume that this measurement was indeed a peak AS. In this case, no optimum temperature for AS could be determined. We prioritised studies, which acclimated their fish. We avoided taking AS where fish were acutely exposed to a range of temperatures.

#### (b) Latitudinal and life history data

Latitudinal range data were available in FishBase (Froese and Pauly, 2014) and metabolic rate data for 116 fish species were taken from the literature. Only five species for which data were available, *Pagothenia borchgrevinki*, *Sillaginodes punctatus*, *Colossoma macropomumin*, *Alcolapia graham* and *Bidyanus bidyanus* were not included in the analyses, leaving 111 species. They were excluded from the analysis because they were designated as southern hemisphere species, and this hemisphere is thermally less variable than the northern hemisphere (Sunday *et al*., 2011, see Fig. 1b), thus species distributions within the two hemispheres are not thermally comparable. Absolute latitude range of each species, calculated as the highest latitude minus the lowest latitude at which a species is found, was used in this analysis as an indicator of the current distribution range, referring to a range from 0° to 90°. For any species with a distribution range overlapping the equator (e.g. 64°N—18°S; n = 39 species), we considered the absolute latitude range expanding from the equator up to the maximum latitudinal point of the species' range in the northern hemisphere (in the example, 64°). Tropical, temperate and polar species are known to cover different distribution ranges according to the CVH and Rapoport's rule. Therefore, the mid-latitude position for each of 111 fish species was calculated from the average of the distribution range (considering northern hemisphere values as positive and southern hemisphere values as negative) and based on this, each species was categorised as either tropical (0–20°), temperate (20–60°) or polar (> 60°). We also collected information from FishBase on lifestyle (benthic, benthopelagic, pelagic or reef-associated), habitat (marine, freshwater or marine/freshwater anadromous and diadromous species) and behaviour (migratory or sedentary species) for all species included in the analyses, to account for these factors. We considered migratory species to be those that undergo active migrations for reproductive and/or feeding purposes.

#### (c) Calculating thermal breadth aerobic scope

From our data set of 111 species, we selected species for which AS data were tested at three or more different temperatures and the AS thermal reaction norm could be fit to a Gaussian model with three parameters (thermal peak AS, *T*_opt_ and b; n = 32 species). For each species, this model was a bell-shaped curve with *T*_opt_ (optimal temperature) taken as the temperature at which AS peaks. We then calculated the range of temperatures at which AS remains above the 90% threshold (Anttila *et al*., 2014; Farrell, 2016). As the thermal range within which AS is above 90% of peak AS is arbitrary, we also calculated the range of temperatures at which AS remains above 80, 75 and 60% of peak AS.

### Data analysis

Statistical models of absolute latitudinal range were generated with the phylogenetic generalised least squares (PGLS) method (Grafen, 1989; Garland and Ives, 2000) using the caper package (Orme, 2013) in R (version 3.3.0 R Foundation for Statistical Computing). The statistical significance level of all tests was set at p = 0.05. The phylogenies for the fish species in the analyses were generated from the ‘Open Tree of Life’ (see Appendix 1, Supplementary Fig. S1.1) (Hinchliff *et al*., 2015) using the ‘rotl’ package (Michonneau *et al*., 2016). A measure of phylogenetic correlation, λ, was estimated by fitting PGLS models with different values of λ to find the value that maximises the log likelihood. Lambda is the degree to which trait evolution deviates from a ‘Brownian motion’ model (traits evolving by the accumulation of small, random changes over time), and thus provides a measure of the degree of phylogenetic correlation in the data (Freckleton *et al*., 2002). Lambda = 1 retains the Brownian motion model, indicating that the trait covariance between any two species is directly proportional to their shared evolutionary history, while lambda = 0 indicates phylogenetic independence (the trait values across species are entirely unrelated to the phylogeny of those species). Intermediate lambda values indicate that trait evolution is phylogenetically correlated but less than expected under the Brownian motion model (for more details, see appendix A of Halsey *et al*., 2006). All variables in the model were centered prior to analysis.

#### (a) Absolute latitudinal range model for peak AS

The following PGLS model was fitted to 111 species:

$$\begin{align*} {ALR}_i&={\beta}_0+{\mathrm{\beta}}_1{\log}_{10}\left({AS}_i\right)+{\beta}_2{\log}_{10}\left({mass}_i\right)\\&\quad+{\beta}_3{temperature}_i+{\beta}_4\mathrm{I}\left({lifestyle}_i= benthopelagic\right)\\&\quad+{\beta}_5\mathrm{I}\left({lifestyle}_i= pelagic\right)+{\beta}_6\mathrm{I}\left({lifestyle}_i= reef\right)\\&\quad+{\beta}_7\mathrm{I}\left({habitat}_i= marine\right)\\&\quad+{\beta}_8\mathrm{I}\left({habitat}_i= marine\& freshwater\right)\\&\quad+{\beta}_9\mathrm{I}\left({behaviour}_i= sedentary\right)\\&\quad+{\beta}_{10}\mathrm{I}\left({thermalgroup}_i= temperate\right)\\&\quad+{\beta}_{11}\mathrm{I}\left({thermalgroup}_i= polar\right)\\&\quad+{\beta}_{12}{\log}_{10}\left({AS}_i\right)\mathrm{I}\left({thermalgroup}_i= temperate\right)\\&\quad+{\beta}_{13}{\log}_{10}\left({AS}_i\right)\mathrm{I}\left({thermalgroup}_i= polar\right)+{\varepsilon}_i \end{align*}$$

where the response variable was the absolute latitude range from 0° to 90° of the $i$th species (${ALR}_i$). The continuous predictor variables were log_10_ (AS in mg O_2_ h^−1^), log_10_ (mass in g) and the acclimation temperature (°C) at which AS was measured, and the categorical predictor variables were lifestyle (benthic, benthopelagic, pelagic, reef associated), habitat (freshwater, marine, freshwater/marine), behaviour (migratory, sedentary) and thermal group (tropical, temperate, polar). AS and mass were log_10_-transformed to homogenize variance in absolute latitudinal range with respect to these variables. Log_10_-transformed mass was included as a covariate in the model because it has scaling effects on metabolic rate data, such as AS (Gillooly *et al*., 2001). Including log-transformed mass in an additive regression model containing log_10_(AS) has the effect, on the exponential scale, of flexibly adjusting the AS regression coefficient for the expected multiplicative effects of mass on AS, in addition to other potential effects of mass on latitudinal range not mediated through AS. Temperature was included in the model because it influences metabolic rate. All the following variables can impact latitudinal range sizes and were therefore also included in our PGLS model: lifestyle (benthic, benthopelagic or pelagic), habitat (freshwater, marine or marine/freshwater combination), behaviour (migratory or sedentary) and thermal group. Different lifestyles, habitats (Rohde *et al*., 1993) and behavioural strategies can either constrain or favour the dispersal capacity of a species and therefore limit or increase its ability to expand its distribution range. The thermal group of a species can affect its distribution range according to Rapoport's rule (Stevens, 1989), which states that a decrease in latitudinal distribution range can be observed towards the equator, implying that tropical species tend to have smaller distribution ranges (Stevens, 1989). Furthermore, daily, seasonal and annual temperature variations differ in magnitude depending on latitude of occurrence, with high thermal fluctuations at temperate latitudes and low thermal variations at tropical and polar latitudes (Sunday *et al*., 2011). Thus, thermal group was included in the model to account for thermal variability across latitudes. Thermal history experienced by a species affects all physiological processes such as AS, and depends on thermal group; consequently, we allowed for the possibility that the effect of AS might vary by thermal group by including an interaction term between these two variables. In total, 14 fixed effects were estimated, including the intercept, ${\beta}_0$, 11 main effects, ${\beta}_{1-11}$, and two interaction effects, ${\beta}_{12}$ and ${\beta}_{13}$. Finally, ${\varepsilon}_i$ represents the residual error of the $i$th species. The residual errors were assumed to be drawn from a normal distribution with mean zero and a variance–covariance matrix with a structure that allows phylogenetically close species to covary to an extent determined by λ.

#### (b) Absolute latitudinal range model for thermal breadth AS

PGLS analysis was also used to explore the relationship between absolute latitudinal range and 90% of thermal breadth of AS in 32 species (see Appendix 2, Fig. S2.1, Fig. S3.1-S3.3). The model was:

$$ {ALR}_i={\beta}_0+{\beta}_1{TBAS}_i+{\beta}_2{\log}_{10}\left({mass}_i\right)+{\varepsilon}_i $$

where the absolute latitudinal range of the $i$th species (${ALR}_i$) was modelled as the sum of an intercept, ${\beta}_0$, the effects of thermal breadth of AS (TBAS) and log_10_ (mass in g), modelled by ${\beta}_1$ and ${\beta}_2$, respectively. Due to the smaller sample size of 32 species, to avoid overfitting, only two predictor variables were included in the model (Harrell Jr, 2015). The residual errors, ${\varepsilon}_i$, were modelled as in the model for peak AS. We performed models for other thresholds of thermal breadth AS (80%, 75% and 60%, see suppl.material).

## Results

### (a) Is latitudinal range predicted by peak AS?

Overall, our model explained 36.3% of the observed variation in absolute latitudinal range for 111 fish species, but log peak AS was a non-significant main effect (Table 1; t = −0.35, p = 0.725). The model indicated that lifestyle strategies do not influence range distribution (Table 1, Fig. 3a). Furthermore, the model showed that species spending at least part of their life cycle in marine habitats have wider absolute latitudinal distributions than do entirely freshwater species (*t*_marine_ = 4.37, *P* < 0.001, *t*_marine/freshwater_ = 3.09, *P* = 0.003, Fig. 3b), while sedentary species tend to have smaller absolute latitudinal ranges than do migratory species (*t*_sedentary_ = −1.106, *P* = 0.272, Fig. 3c.). There was a significant interaction between log peak AS and thermal group: temperate species showed a negative relationship between AS and absolute latitudinal range (*t* = −2.42, *P* = 0.02), while tropical and polar species exhibited no relationship (Fig. 2). The slope value for temperate species was −5.48 (95% CI, −10.32 to −0.64). Thus, temperate species exhibit an estimated 5.48° decrease in absolute latitudinal range distribution for each 10-fold increase in peak AS. Lambda for the model was negligible (λ = 0.00), suggesting no phylogenetic inertia in trait covariance.

**Figure 4:**
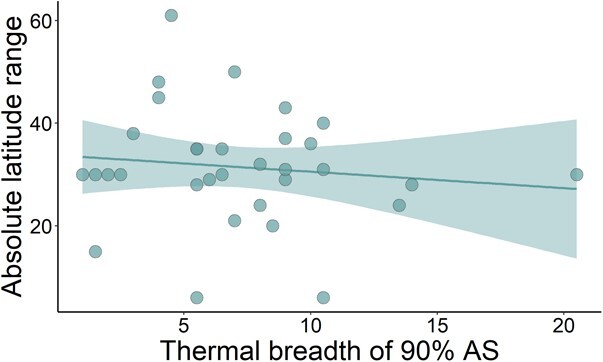
There is no discernible relationship between absolute latitudinal range and thermal breadth of 90% aerobic scope for 32 fish species. Each data point represents a distinct species. The shaded area represents 95% confidence intervals around the line of best fit.

### (b) Is latitudinal range predicted by thermal breadth AS?

The PGLS model including the thermal breadth of AS explained just 5.9% of the variation observed in the latitudinal range of 32 species. Lambda was negligible (λ = 0.00), suggesting no phylogenetic inertia in trait covariance. There was no relationship between 90% of peak AS thermal breadth on latitudinal range (*t* = −0.83, *P* = 0.41, Fig. 4, Table 2). This was also the case for the calculated thresholds 80% and 60% AS thermal breadths (see Tables S3.2 and S3.4, Fig. S3.1 and S3.3). However, at the 75% threshold, there was a negative relationship between AS breadth and absolute latitude range (Table S3.3, Fig. S3.2).

**Table 2:** Summary of the PGLS model testing for the effects on absolute latitude range (0–90°) of thermal breadth of aerobic scope (thermal 90% AS (mg O_2_ h^−1^)) and mass (log_10_ g). *R^2^* = 0.059 F_2,29_ = 0.905, p = 0.416, n = 32 species, λ = 0.00, d.f. = 29

Term	Estimate	s.e.	*t*	*p*
Intercept	30.47	4.93	6.18	<0.001
Thermal 90% AS	−0.42	0.5	−0.83	0.41
Log_10_ mass	2.63	2.23	1.18	0.25

## Discussion

Current theory implies a positive correlation between aerobic capacity in ectotherms, such as fishes, and their geographic distribution (Naya and Bozinovic, 2012). Contrary to these expectations, however, in teleost fish, we observed no relationship between thermal breadth aerobic scope (AS) and latitudinal range in teleost fish, and a *negative* relationship between peak AS with interaction of latitudinal midpoint and latitudinal range, in temperate species (Fig. 2). Thus, while peak AS did not explain latitudinal range in either polar or tropical species, in temperate species those with a high AS showed a lower-latitude range than did those with a low AS.

The data here indicate that a greater aerobic capacity (both in terms of AS peak and the breadth of temperatures across which AS is high) does not convey an advantage for a fish species to inhabit a wider geographical range (Fig. 1). This contradicts the CVH, which states that high-latitude species have broader thermal tolerance and greater physiological plasticity due to the thermal fluctuations they experience. However, this hypothesis has mainly been studied in terrestrial ectotherms (Naya and Bozinovic, 2012) and has limited evidence for aquatic species. Furthermore, according to Rapoport's rule, range distributions should be smaller in species that occur at lower latitudes (Stevens, 1989). However, this rule was originally applied to terrestrial species, with mixed evidence of it applying to fishes (Rohde *et al*., 1993; Rohde and Heap, 1996). Similarly, in the current study, we observed no significant effect of thermal group (temperate, tropical or polar) on the latitudinal range of species. Our data thus support the view that Rapoport's rule (Stevens, 1989) may only apply within specific latitudinal ranges and biogeographical contexts.

In our study, the AS data came from studies that mostly measured AS at one acclimation temperature (58 of 106 studies). Due to this, we cannot presume that these AS data correspond to the thermal peak AS. In the wild, species may rarely utilise their entire aerobic capacity. Our results might display a trend of the influence of AS on distribution ranges in 111 fish species.

It is possible that, within a species, local adaptation in populations across a broad geographical range could result in specialisation to cope with regional thermal conditions. A species might also cope with thermal extremes across its range by decreasing its activity and feeding, particularly during seasonal thermal shifts, allowing it to occupy a wide thermal and geographical range despite having a relatively low AS. This is a strategy of many temperate freshwater species and at least some temperate marine species during overwintering (Ultsch, 1989). Reducing activity during thermal extremes may be less viable for tropical species, however, because they are exposed to relatively high temperatures even during the coolest parts of the year, and so must possess a large AS for activity and other physiological processes year-round (Nati *et al*., 2016). This could be one reason why the negative association between AS and geographical range is attenuated in tropical species. Species with a narrower distribution may evolve greater specialisation to living within a defined range of environmental parameters with fewer functional tradeoffs, perhaps permitting a larger AS. In 92 fish species, for example, increased AS is generally accompanied by an increase in energetic maintenance costs and energetic demand even at rest (Killen *et al*., 2016). Species that are specialised for relatively constant thermal regimes may be able to circumvent this fundamental trade-off to some extent therefore reducing the costs of an increased AS. We note, however, that we were able to find suitable data on far fewer tropical species than temperate species, which may have influenced our results. Additionally, there is a lack of physiological data for fish species inhabiting the southern hemisphere (Seebacher *et al*., 2015). It is also possible that migratory species are able to track their optimal or preferred thermal niches during migrations. There are currently insufficient data on the migratory patterns of species and the thermal conditions they experience to have included this factor in our analyses, but it is an important area for future research.

The fact that peak AS relates to distribution range according to thermal group (temperate), and that breadth of AS does not influence distribution range, implies that fish adjust AS according to energetic needs and environmental conditions (Norin *et al*., 2016). It should be noted that sample size for the thermal breadth of AS was relatively low while the variation in the data was considerable. While this may have contributed to the lack of an observed effect, the parameter estimates indicate that any effect of thermal breadth would nonetheless be extremely small. Species or individuals can have varying degrees of plasticity in AS allowing them to cope with environmental stressors (Munday *et al*., 2012, 2013; Norin *et al*., 2016). One threshold breadth (75%) had a negative association with the absolute latitudinal range. It seems that at this breadth, species display a trade-off between maintaining this breadth and spreading their range. They might favour nearby food resources rather than expanding their range. The current study indicates that despite the plasticity of AS, species or individuals do not necessarily need to have a high AS to function efficiently over a larger range of environmental temperatures (Nati *et al*., 2016). Furthermore, other characteristics, such as lifestyle, body size, trophic level and fecundity, can all interact to influence the distribution of species and possibly outweigh any direct effect of AS on distribution. Fishes with different lifestyles (benthic, benthopelagic and pelagic) have different energy requirements and constraints (Killen *et al*., 2016), dispersal capacities and migration patterns (Eliason *et al*., 2011; Demer *et al*., 2012). Benthic fish species are known to have a lower AS than pelagic species, giving them less aerobic capacity to direct toward dispersal (Killen *et al*., 2016). Furthermore, benthic species might be less constrained by a reduction in AS due to their less active lifestyle. Here, lifestyle was not a predictor for latitudinal distribution ranges. Further, different life stages might be more vulnerable than others. Larvae, eggs and spawning adults are believed to be the most affected (Dahlke *et al*., 2020, Pörtner and Farrell, 2008). In our data set, we had juveniles and adults, the life stages that are the more robust in term thermal challenges. Another area for additional targeted research would be the effects of life stage on the interplay between aerobic capacity and distribution. Although body mass appears to be the primary driver of changes in aerobic capacity during ontogeny both within and among species (Killen *et al*., 2007; Killen *et al*., 2016), behavioural differences between life stages (e.g. juvenile versus adult) could affect the proportion of aerobic scope remaining for species after factors such as activity are accounted for.

Additionally, other than AS, fishes, populations and individuals can vary in their thermal limits (CT_max_). CT_max_ set the upper latitude boundaries. We know that tropical not only have the highest CT_max_ but also have the lowest intraspecific variation in their CT_max_ (Nati *et al*., 2021). This makes tropical species less resistant to future warming events.

In conclusion, we found evidence that peak AS is negatively related to the geographical distribution of temperate teleost fish, suggesting that greater AS can be a constraint in this regard. Maintaining their maximum aerobic capacity is believed to come with an energetic cost. It has been suggested that fish species distributions may be linked to the thermal sensitivities and limits of mitochondrial stability and functioning of the heart (Iftikar *et al*., 2014).

## Funding

This work was supported by an Aides à la Formation Recherche doctoral grant from the Fonds National de la Recherche Luxembourg (4005263), the Natural Environment Research Council Advanced Fellowship (NE/J019100/1) and the European Research Council Starting Grant (640004).

## Conflict of Interest statement

The authors declare no competing interests.

## Data availability

Data supporting the results of this study are available in this manuscript and its supplementary files.

## Authors' Contributions

Conception: J.J.H.N., J.L., S.S.K.; data collection: J.J.H.N., S.S.K.; data analysis: J.J.H.N., L.H., P.C.D.J., S.S.K.; manuscript writing: J.J.H.N.,. S.S.K.; manuscript reviewing: J.J.H.N., L.H., PC.D..J., J.L., S.S.K.

## Supplementary Material

Web_Material_coad012
