# Supplementary material for: Does aerobic scope influence geographical distribution of teleost fishes?
Source: Conserv Physiol. 2023 Mar 29;11(1):coad012. doi: 10.1093/conphys/coad012 (PMC10061159; doi:10.1093/conphys/coad012)
Supplement: Web_Material_coad012 [file web_material_coad012.zip › Supplementary material08.11.22.pdf]

Supplementary information

APPENDIX 1

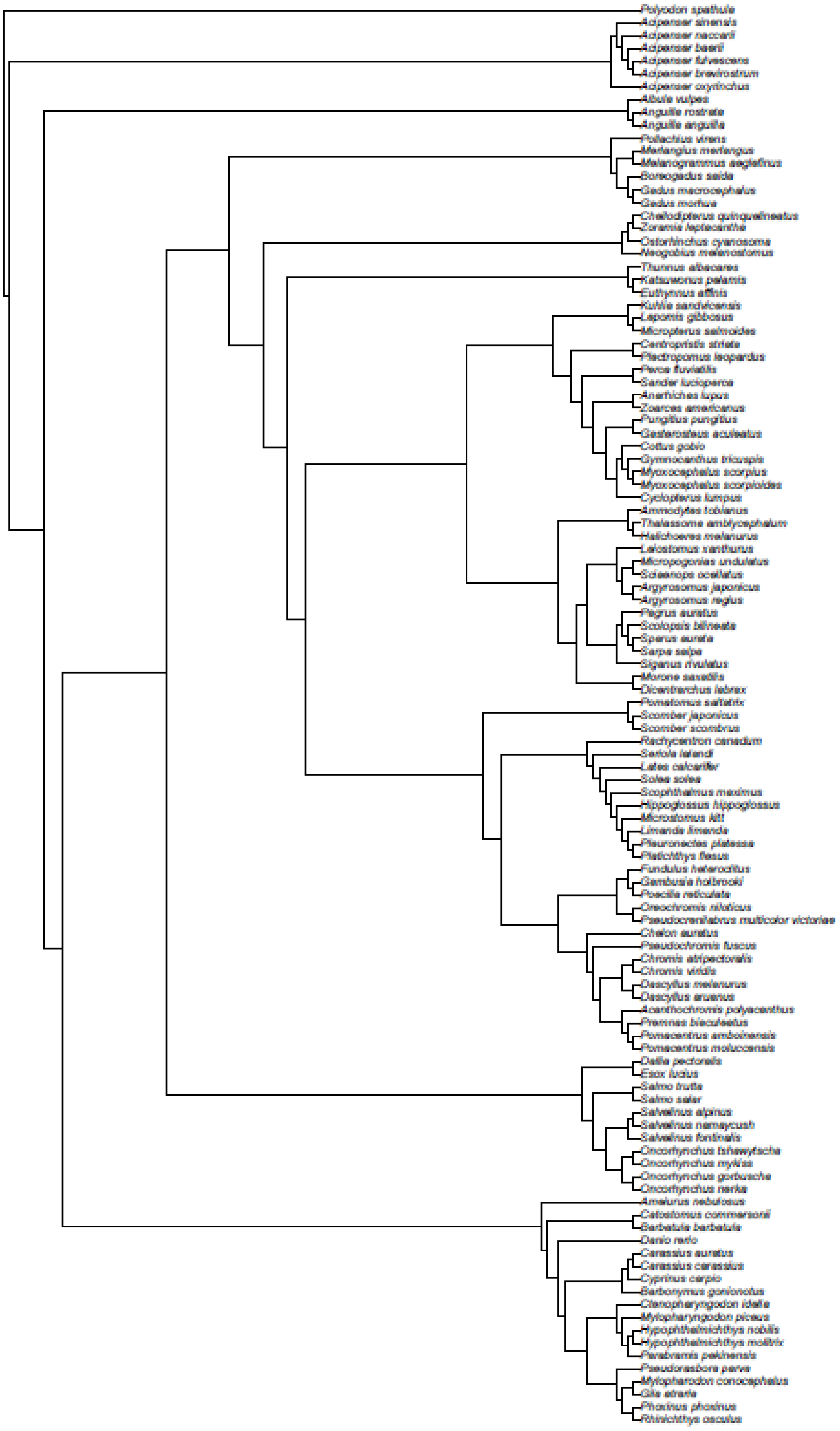

Figure S1.1 Phylogenetic tree generated on 111 fish species used in PGLS absolute latitude range model. Branch lengths represented in millions of years.

APPENDIX 2

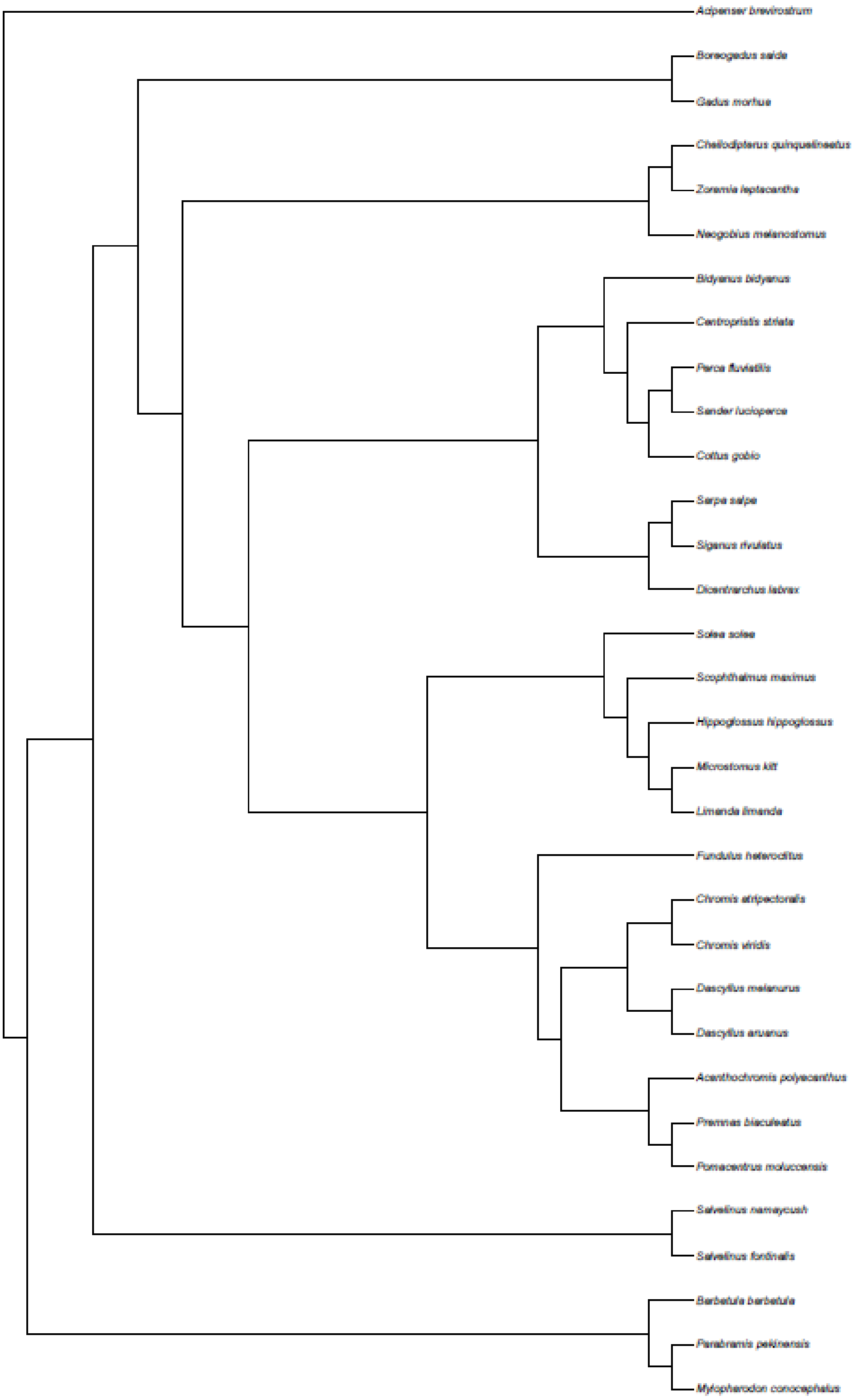

Figure S2.1 Phylogenetic tree generated on 32 fish species used in PGLS absolute latitude range model. Branch lengths represented in millions of years.

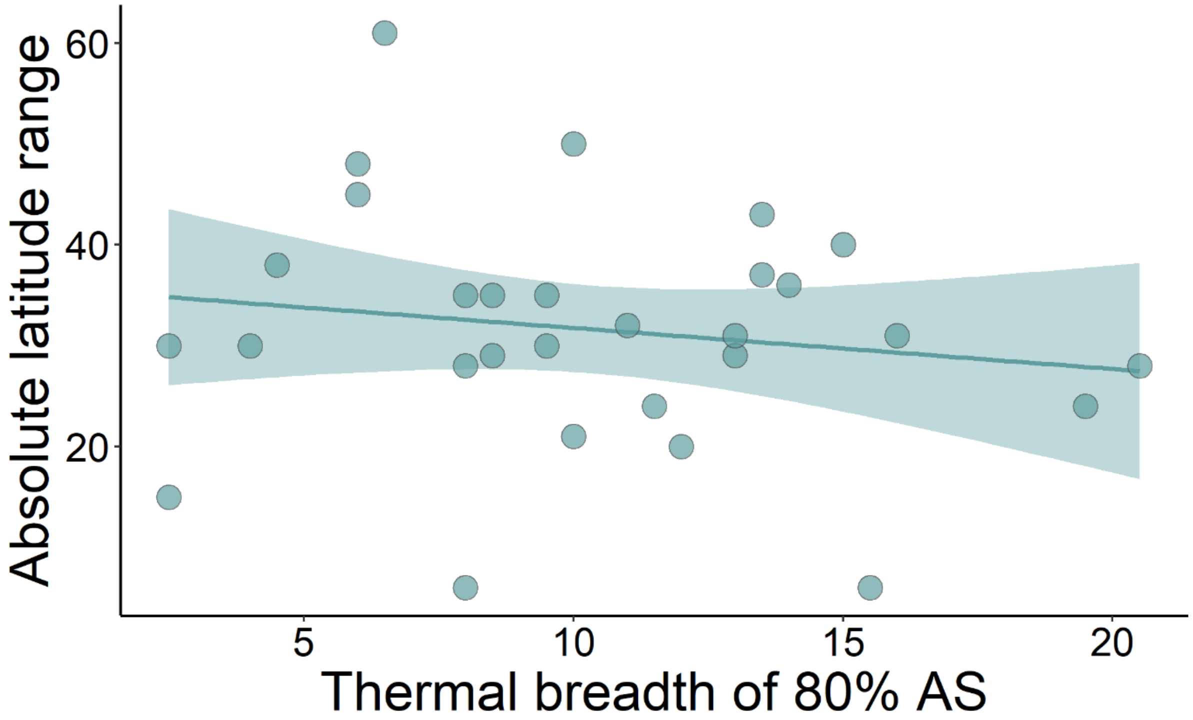

Figure S3.1. There are no discernible relationships between absolute latitudinal range and thermal breadth of 80% aerobic scope for 29 fish species. Each data point represents a distinct species. Shaded areas represent 95% confidence intervals around the lines of best fit.

Table S3.2 Summary of the PGLS model testing for the effects on absolute latitude range (0-90°) of thermal breadth of aerobic scope (thermal 80% AS (mg O_2 h^{-1})) and mass (log_{10} g). R^2 = 0.076, $F_{2,26}$ = 1.067, p = 0.359, n = 29 species, λ = 0.00, d.f. = 26.

term	estimate	s.e.	*t*	*p*
Intercept	32.02	6.35	5.043	<0.001
thermal 80% AS	-0.52	0.497	-1.05	0.30

log_{10} mass	3.09	2.58	1.2	0.24

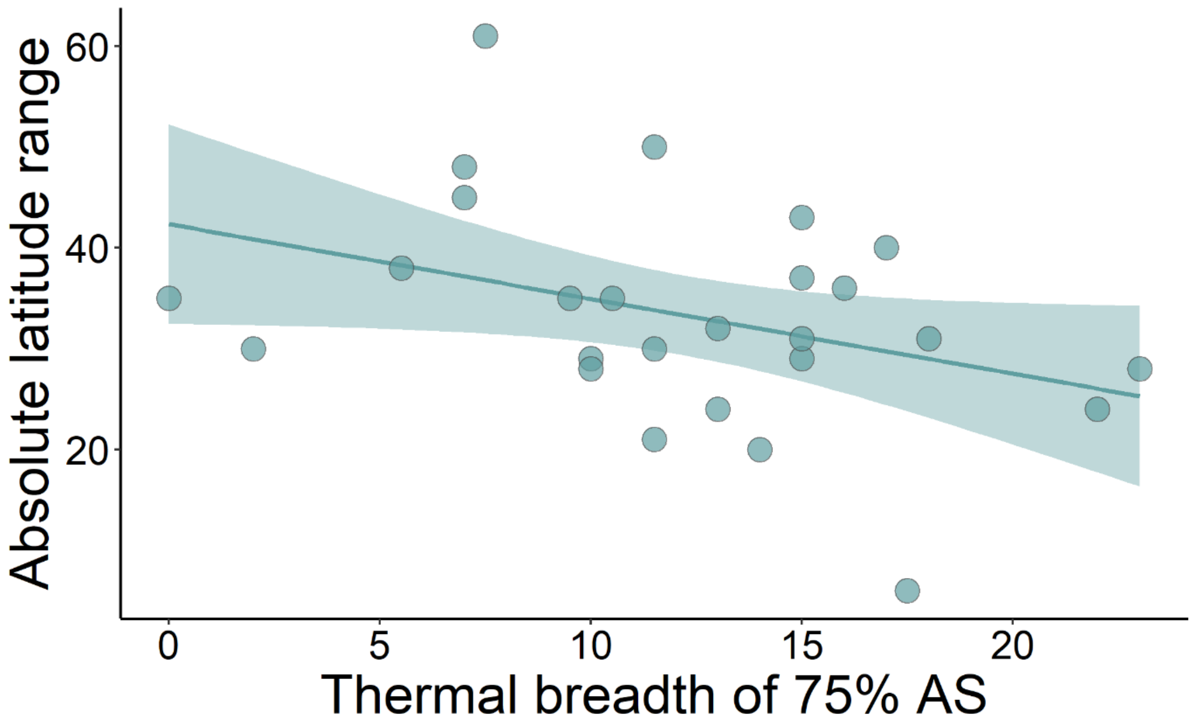

Figure S3.2. There are no discernible relationships between absolute latitudinal range and thermal breadth of 75% aerobic scope for 26 fish species. Each data point represents a distinct species. Shaded areas represent 95% confidence intervals around the lines of best fit.

Table S3.3 Summary of the PGLS model testing for the effects on absolute latitude range (0-90°) of thermal breadth of aerobic scope (thermal 75% AS (mg O_2 h^{-1})) and mass (log_{10} g). R^2 = 0.249, $F_{2,23}$ = 3.812, p = 0.037, n = 26 species, λ = 0.00, d.f. = 23.

**term**	**estimate**	**s.e.**	***t***	***p***
Intercept	36.03	5.9	6.11	<0.001
thermal 75% AS	-0.80	0.36	-2.21	0.037
log_{10} mass	4.26	2.31	1.85	0.08

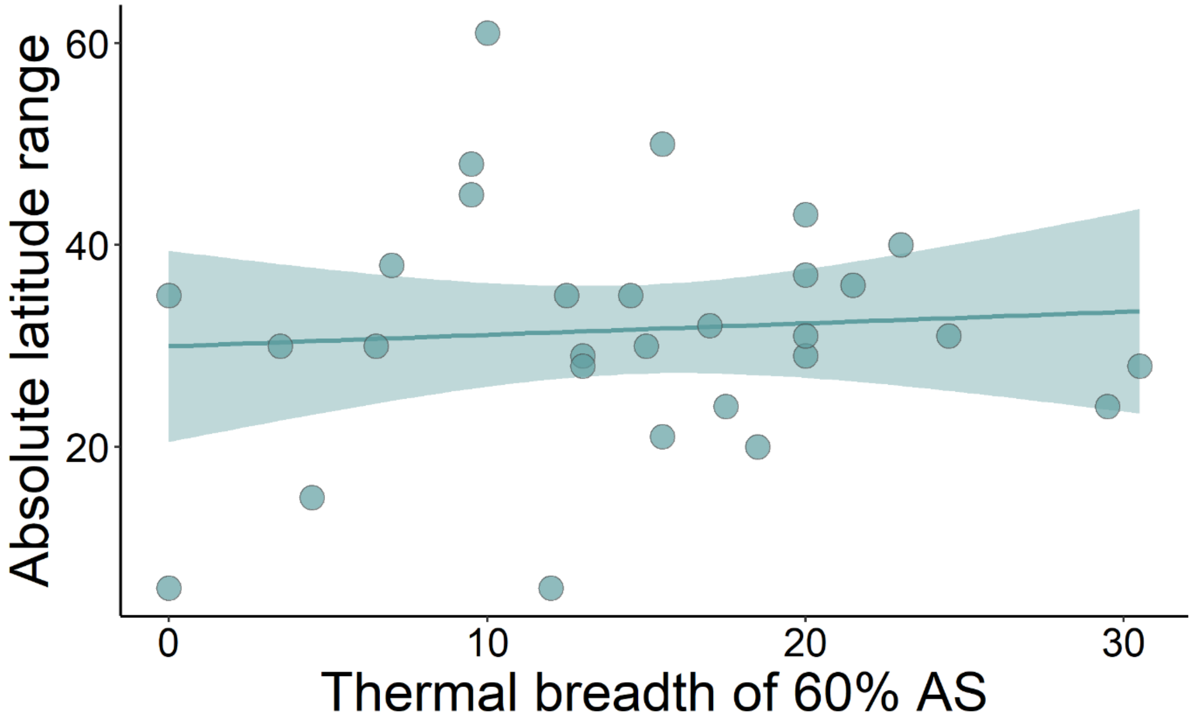

Figure S3.3. There are no discernible relationships between absolute latitudinal range and thermal breadth of 60% aerobic scope for 29 fish species. Each data point represents a distinct species. Shaded areas represent 95% confidence intervals around the lines of best fit.

Table S3.4 Summary of the PGLS model testing for the effects on absolute latitude range (0-90°) of thermal breadth of aerobic scope (thermal 60% AS (mg O_2 h^{-1})) and mass (log_{10} g). R^2 = 0.038 $F_{2,126}$ = 0.507, p = 0.608, n = 29 species, λ = 0.00, d.f. = 26.

**term**	**estimate**	**s.e.**	***t***	***p***
Intercept	26.85	5.89	4.56	<0.001
thermal 60% AS	0.05	0.3	0.18	0.86
log_{10} mass	2.45	2.64	0.93	0.36
